# Of Mice and Fungi: *Coccidioides* spp. Distribution Models

**DOI:** 10.3390/jof6040320

**Published:** 2020-11-27

**Authors:** Pamela Ocampo-Chavira, Ricardo Eaton-Gonzalez, Meritxell Riquelme

**Affiliations:** 1 Department of Microbiology, Centro de Investigación Científica y Educación Superior de Ensenada (CICESE), Ctra. Ensenada-Tijuana No. 3918, Ensenada, Baja California 22860, Mexico; pamela.ocampo@outlook.com; 2 Academic Unit of Ensenada, Universidad Tecnológica de Tijuana, Ctra. a la Bufadora KM. 1, Maneadero Parte Alta, Ensenada, Baja California 22790, Mexico; eaton.gonzalez@uttijuana.edu.mx

**Keywords:** *Coccidioides* spp., distribution modeling, Maxent, GIS, biological variables

## Abstract

The continuous increase of Coccidioidomycosis cases requires reliable detection methods of the causal agent, *Coccidioides* spp., in its natural environment. This has proven challenging because of our limited knowledge on the distribution of this soil-dwelling fungus. Knowing the pathogen’s geographic distribution and its relationship with the environment is crucial to identify potential areas of risk and to prevent disease outbreaks. The maximum entropy (Maxent) algorithm, Geographic Information System (GIS) and bioclimatic variables were combined to obtain current and future potential distribution models (DMs) of *Coccidioides* and its putative rodent reservoirs for Arizona, California and Baja California. We revealed that *Coccidioides* DMs constructed with presence records from one state are not well suited to predict distribution in another state, supporting the existence of distinct phylogeographic populations of *Coccidioides*. A great correlation between *Coccidioides* DMs and United States counties with high Coccidioidomycosis incidence was found. Remarkably, under future scenarios of climate change and high concentration of greenhouse gases, the probability of habitat suitability for *Coccidioides* increased. Overlap analysis between the DMs of rodents and *Coccidioides*, identified *Neotoma lepida* as one of the predominant co-occurring species in all three states. Considering rodents DMs would allow to implement better surveillance programs to monitor disease spread.

## 1. Introduction

Coccidioidomycosis (CM), a reemerging disease also known as Valley Fever, is endemic to arid and semi-arid regions of the American continent. Southern Arizona and California in the United States, and Sonora, Nuevo Leon, Coahuila and Baja California in Mexico, are considered important endemic regions based on the high prevalence of the disease they present [1]. The high incidence rate values of CM reported during the last decade in Arizona and California (https://www.cdc.gov/fungal/diseases/coccidioidomycosis/statistics.html), represent a great concern both in terms of public health and economically [2].

*Coccidioides immitis* and *C. posadasii* are the only known fungal species of the genus responsible for causing CM. *Coccidioides* spp. distribution in soils is very irregular, and this hinders the detection of positive sites even in highly endemic areas [1,3]. Great efforts to characterize *Coccidioides* habitat and elucidate the basis for this scattered distribution have been made. However, most of the information on the environmental preferences of *Coccidioides* was explored in the 1950s and 1960s, hence there is a need to obtain current data to better understand this distribution [2]. Alkaline, sandy-textured and high-salinity soils, have been correlated with the presence of *Coccidioides* spp. [4,5]. In addition to these abiotic micro-environmental variables, the incidence of the disease has been correlated with abiotic macro-environmental variables such as rainfall, drought, warm surface air temperatures and dry soils [6,7,8]. Baja California in Mexico, and California and Arizona in the United States, all share similar climate types (Arid and Semiarid), and similar periods of precipitation followed by some periods of drought [9]. In all three states, CM outbreaks have been linked to drought periods [6,7].

Analysis of aerial observations from 1948 to 2008 and climate model simulations for the period of 1948–2100, showed that dry lands have expanded globally in the last years as a result of climate change, and predict that it will continue to expand [10]. Cumulative scientific evidence shows that climate change is affecting living systems [11,12]. Global meta-analyses have been carried out on over 1700 species, showing distribution range shifts by species of birds, butterflies and alpine herbs averaging 6.1 km per decade towards the poles and advancement of spring events in species of herbs, shrubs, trees, birds, butterflies and amphibians by 2.3 days per decade, implicating climate as an important driving force in natural systems [13,14]. These registered shifts are a subject of concern for the future. Several attempts to model species distribution considering a future scenario of climate change have been carried out using projected bioclimatic variables and greenhouse gas emissions from representative concentration pathways (RCPs) for twenty to fifty years from now and the species geographic range increases or decreases depending on the species ecology. These changing scenarios can be investigated through some of the emerging variables as a consequence of climate change. Drought and rainfall intensities increase as a consequence of climate change and this can influence microbial activities such as respiration and growth [15,16]. As a driver force, drought could shape the composition of the microbial community and fungi could respond in a sensitive, tolerant or opportunistic way [16,17].

At the biotic level, animals that become infected (yet do not necessarily develop the disease), such as bats, armadillos and rodents, could act as reservoirs during the life cycle of *Coccidioides* spp. [18]. Studies in Kern County, California, and San Carlos, Arizona, have detected *Coccidioides* spp. in soils with rodent activity [19,20]. Moreover, buried corpses of infected pets such as cats and dogs become organic matter susceptible to be decomposed by the fungus that continues its life cycle in soils [2]. Genomic analysis of *Coccidioides* suggests that it has adapted to life within an immunocompetent mammalian host, and it has more genes involved in animal infection, both in colonization and survival (proteases such as keratinases), than genes involved in plant infection, such as enzymes responsible for cell wall degradation [21], which reinforces the importance of animal hosts in *Coccidioides* life cycle. This could explain why greater positive samples are obtained from soils rich in organic matter derived from animal carcasses, rather than soils lacking animal activity [18].

Valle de las Palmas in Tecate, Baja California, Mexico, was previously identified as an endemic site for CM, based on reports of acquired CM by individuals that had visited the site, and on positive soil isolation of *C. immitis* [22]. Subsequently, *Coccidioides* spp. was detected from soil samples of that site by using a nested polymerase chain reaction (PCR) approach [1]. Higher prevalence of *Coccidioides* spp. was found inside burrows than in topsoil, suggesting that the microenvironment inside the burrows may favor the presence of *Coccidioides* spp. and maintain the stability of the fungal community structure [23]. To further investigate the relationship between *Coccidioides* spp. and small rodents, serum extracted from four species of rodents captured in Valle de las Palmas was tested by the enzyme-linked immunosorbent assay (ELISA) [24]. From 40 serum samples, coccidioidal antibodies were detected in two samples corresponding to *Peromyscus maniculatus* (Deer mouse) and one corresponding to *Neotoma lepida* (Desert woodrat). No antibodies were detected in *Chaetodipus fallax* (San Diego pocket mouse) and *Dipodomys simulans* (Dulzura kangaroo rat) samples, even though the latter species correspond to genera reported earlier as presenting evidence of infection by *Coccidioides* spp. [25]. The unsuccessful detection of *Coccidioides* spp. antibodies in *C. fallax* and *D. simulans* was attributed to the use of secondary antibodies raised in the common mouse (*Mus musculus*), which belong to a family phylogenetically distant to the Heteromyidae (where *C. fallax* and *D. simulans* belong to)**, but close to the Cricetidae (where *N. lepida* and *P. maniculatus* belong to).

Species distribution models (SDM), in particular Environmental Niche Models, aim to estimate the environmental conditions that are suitable for a species by associating known species occurrence records with environmental variables that are expected to affect the species probability of persistence [26]. The result is projected on a map composed of pixels, which represent the probability values of habitat suitability [27]. Areas with high probability can be translated to environmentally suitable areas, where the species could occur. SDMs are considered an emerging tool for modeling fungal distribution, particularly of pathogenic fungi [28]. However, development of SDMs for several fungal species could face some limitations, such as ineffective detection methods, biased sampling and the difficulty of finding environmental covariates at appropriate spatial and temporal scales [28]. Previously, the potential distribution of *Coccidioides* spp. was modeled using GARP (Genetic Algorithm for Rule Set Production), 18 occurrence points, 11 climatic variables as well as a digital elevation model in adjoining states of the Mexico–USA border [29]. Geo-referenced clinical records had a good match with probable areas of habitat suitability for *Coccidioides* spp., based on the distribution model (DM) generated [29]. GARP outputs are binary predictions and although the conversion of SDMs output from continuous to binary predictions is widely used for many ecological, biogeographical and conservation applications, this conversion may lead to a loss of information [30]. Therefore, we modeled *Coccidioides* distribution using Maxent, whose outputs are continuous. This type of output allowed us to identify more precise distinctions between the modeled suitability of different areas. Additionally, we used more occurrence data as well as selected environmental data.

The aim of this work was to obtain DMs for *Coccidioides* spp. with more delimited and identifiable suitable areas using current bioclimatic variables and those projected for 2070 at the highest Representative Concentration Pathway (RCP) value considered by the Intergovernmental Panel on Climate Change (IPCC). In addition, DMs for *C. fallax, C. penicillatus, D. simulans, D. merriami, N. lepida* and *P. maniculatus*, previously reported as putative rodents’ reservoirs for the fungus altogether in the endemic states of California and Arizona in the United States, and Baja California in Mexico, were obtained and compared to *Coccidioides* spp. DM, in order to find geographic overlap of habitat suitability, which would reinforce the possible biological relationship between the species.

## 2. Materials and Methods

Species DMs were generated for *Coccidioides* spp., *C. fallax*, *C*. *penicillatus, D. simulans*, *D. merriami, N. lepida* and *P. maniculatus* using Maxent version 3.3.3.k with default parameters, other than splitting occurrence points randomly for model calibration and testing (80% and 20%, respectively). In addition, 50 replicates were included using statistical bootstrap to avoid uncertainty. To assess model performance, we used as an indicator the area under the receiver operating characteristic curve (AUC) [31,32].

### 2.1. Occurrence Data

Occurrence data for *Coccidioides* spp. in the United States were provided by B. Barker from the Translational Genomics Research Institute for Arizona, and J. Taylor from the University of California Berkeley for California, and additional occurrence points for California were obtained from published work [19]. Occurrence points correspond to sites, where the fungus was detected by a real-time PCR method [33] and multiplex PCR [19]. Occurrence data for *Coccidioides* spp. in Valle de las Palmas and San José de la Zorra in Ensenada, Baja California, Mexico, correspond to sites where *Coccidioides* was detected by nested PCR in different years and seasons [1,23,24]. However, sampling sites are limited to an area smaller than 1 km^2^, which represent a single pixel from the environmental data used (according to its resolution). Taking into account that Valle de las Palmas and San José de la Zorra have been proposed as positive sites for *Coccidioides* and due to the lack of sampling points that could represent a greater area within these positive sites, we generated 20 random points (10 in Valle de las Palmas and 10 in San José de la Zorra) within two local polygons inside each locality, delimited by their geographic and environmental homogeneity. These data points represent geographic and environmental homogeneous data for these sites and correspond to only a few pixels of the environmental variables used to build the DM. Rodents’ occurrence data was downloaded from GBIF (Global biodiversity information facility). All point data was transformed to .csv files to use as input data on Maxent.

### 2.2. Environmental Data

#### 2.2.1. Current Conditions

A total of 20 environmental variables were used (Supplementary Table S1): 19 bioclimatic variables from WorldClim version 2.0 for the 1970–2000 reference period [34] and sand content from the first 15 cm depth [35]. All environmental data have a 30 arc-second resolution and were clipped to the region of study and transformed to .asc format for processing in QGIS Wien 2.8.

#### 2.2.2. Future Conditions

Climatic variables for future conditions are based on the Representative Concentration Pathway (RCP). This approach provides information on possible development trajectories for the main forcing agents of climate change, including greenhouse gases, with RCP 2.6 being the lowest and RCP 8.5 the highest values [36]. Bioclimatic variables projected for 2070 were built based on the RCP 8.5 scenario (2061–2080 period), downloaded from WorldClim [37], and processed using the same method as for the current conditions, prior to Maxent analysis. The RCP 8.5 is characterized by increasing greenhouse gas emissions over time due to a continuous and growing use of fossil fuels [38,39].

### 2.3. Variable Selection

With the purpose of determining the most important variables for each species, an initial DM for each species was carried out using all 20 environmental variables of current conditions. Subsequently, based on the Jackknife test of variable importance, we chose environmental variables with the highest gain, followed by variables whose contribution was higher than 50% of the initial variable selected, when used in isolation. In addition, variables whose gain decreased the most when omitted were also selected (Supplementary Figure S1). After applying the above criteria, environmental variables that contributed less than 5% were eliminated, with the assumption that if all variables were equally important, each would contribute 5% to the species niche [40]. We picked this criterion to avoid using correlated variables, considering that if two variables are correlated, Maxent automatically assigns a larger contribution to one of them [41]. Environmental variables (Supplementary Table S1) were selected based on the Jackknife test and percentage of contribution, as mentioned previously. To project *Coccidioides* distribution under future scenarios, the same environmental variables were considered.

### 2.4. Match of Suitable Areas for Coccidioides spp. and Rodent Species (Binary Maps)

To visualize where distribution and suitable areas for each species overlap, SDMs were transformed into binary maps, which represent suitable and unsuitable distribution sites for each species. To establish a suitable area, a threshold value was defined by the value of the 10th percentile of training presences, generated by the omission and commission analysis in Maxent [42]. This value reflects the probability value at which 90% of presence records are within the predicted potential area [43]. This threshold has been applied to species with low dispersal power, like the fungus or the rodents of this study that have a small home range. This is considered a conservative threshold, since it does not tend to overestimate the potential distribution area [42,44]. Taking this assumption into account, probability values of the 10th percentile were used as thresholds in QGIS Wien 2.8 to generate binary predictions maps for *Coccidioides* spp., *C. fallax*, *C. penicillatus, D. simulans*, *D. merriami*, *N. lepida* and *P. maniculatus*. For each species DM, all pixel values that are equal to or higher than the threshold receive a value of 1 in a binary map. Pixels with values lower than the threshold would be assigned a value of 0. This reclassification was performed for present and future models. Each of the binary maps for each rodent species was reclassified with discrete values of 1, 10, 100, 1000, 10,000 and 100,000 to differentiate each one respectively, in order to elaborate overlap distribution maps of rodents. These overlapping rodents’ distribution maps were multiplied with the binary maps of the fungus, resulting in maps where the 0 values mean that there is no overlap between the rodent species under study and the fungus. A combination of the assigned discrete values means an overlap between the fungus and one or several of the rodent species.

## 3. Results

### 3.1. Model Performance

Based on Jackknife test results (Supplementary Figure S1A–G), for *Coccidioides* DMs in Arizona, California and Baja California, the most important variables when used individually were mean temperature of warmest quarter (BIO10), precipitation of warmest quarter (BIO18) and isothermality (BIO3), respectively. Variables that decreased gain the most when omitted were precipitation seasonality (BIO15) for Arizona, and minimum temperature of coldest month (BIO6) for both California and Baja California. Jackknife results for rodent models indicated as important variables, when used individually: precipitation of driest month (BIO14), temperature seasonality (BIO4), precipitation of warmest quarter (BIO18) and precipitation seasonality (BIO15) for *C. fallax*, *D. simulans* and *N. lepida* respectively, as well as annual mean temperature (BIO1) for *D. merriami, C. penicillatus* and *P. maniculatus*. Variables that decreased gain the most when omitted were precipitation of driest quarter (BIO17), mean temperature of wettest quarter (BIO8), precipitation of coldest quarter (BIO19) and maximum temperature of warmest month (BIO5) for *C. fallax*, *C. penicillatus, D. merriami* and *D. simulans* respectively, as well as precipitation seasonality (BIO15) for *N. lepida* and *P. maniculatus* (Supplementary Table S1).

To evaluate predictive ability of the DM of a species, we used the area under the receiver operating characteristic curve (AUC), a metric test that assesses whether model predictions are better than random, as well as the DM fitness to true presence and absence data. AUC values range from 0 to 1, with 1 being a model that predicts presences perfectly and 0.5 when predictions are the same as random guesses. Models with an AUC over 0.8 can be considered good and models with an AUC > 0.9 as very good [45].

When using only occurrence points for California and Arizona to obtain *Coccidioides* DM, because of the higher number of presence data points confirmed by molecular techniques, no suitable areas were obtained for Baja California (data not shown). This led us to partition the occurrence data and to generate three DMs for *Coccidioides* using occurrence data from each state independently. For *Coccidioides* DM using occurrence data in Arizona, California and Baja California, AUC values were 0.944, 0.997 and 0.998, respectively (Supplementary Figure S2A). For rodent DM, AUC values were 0.978 for *C. fallax*, 0.904 for *C. penicillatus*, 0.989 for *D. simulans* and 0.905 for *N. lepida* (Supplementary Figure S2B,C,F,G). Even though lower values of AUC were obtained for *P. maniculatus* and *D. merriami* DM (0.800 and 0.811, respectively), they were still considered to have a good predictive ability (Figure S2D,E).

### 3.2. Suitable Habitat for Coccidioides spp. in Arizona, California and Baja California

**Figure 1:**
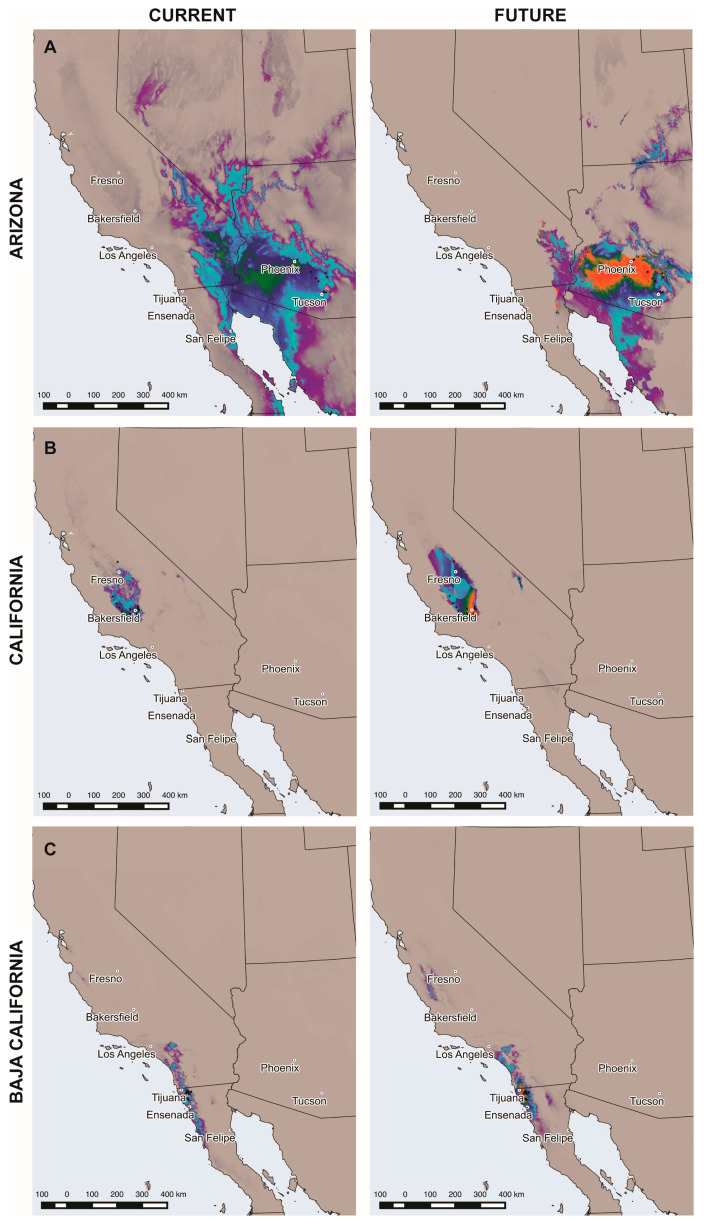
Distribution models for *Coccidioides* spp. Current environmental variables (left), and those projected for 2070 based on a Representative concentration pathway 8.5 (right), were used to model *Coccidioides* spp. distribution, taking into account occurrence points in Arizona (**A**), in California (**B**) and in Baja California (**C**). Habitat suitability for *Coccidioides* spp. is represented in 10 categories based on probability: from 0 to 0.09 (gray), from 0.1 to 0.19 (dark grey), from 0.2 to 0.29 (purple), from 0.3 to 0.39 (turquoise), from 0.4 to 0.49 (dark turquoise), from 0.5 to 0.59 (blue), from 0.6 to 0.69 (dark blue), from 0.7 to 0.79 (green), from 0.8 to 0.89 (orange) and from 0.9 to 1 (red). Black dots represent points where *Coccidioides* spp. has been either identified molecularly or isolated from soil samples.

Using occurrence points for Arizona and current climatic variables conditions (CCVCs) resulted in *Coccidioides* DM with high probability of habitat suitability in Southwest Arizona and Southeast California, with values up to 0.84. For Baja California, suitable areas for *Coccidioides* were predicted along the east coast of the state and in Sonora along the west coast, with higher probability of habitat suitability at the Northwest region of the state (Figure 1A). Using future conditions (RPC 8.5), habitat suitability in all regions showed a more restricted distribution, although with higher probability values of habitat suitability for *Coccidioides*, with values reaching 0.99 (Figure 1A). Using occurrence points from California and CCVCs, the area of habitat suitability of *Coccidioides* DM was projected almost exclusively for that State, in Central Valley between Bakersfield and Fresno, with values up to 0.82 (Figure 1B). A similar pattern of distribution was observed under an RCP 8.5 scenario but with higher values of habitat suitability, reaching values of 0.98 in the east of Bakersfield (Figure 1B). Using occurrence points for Baja California and CCVCs to carry out *Coccidioides* DM, results showed high values of habitat suitability in areas comprising the South of Los Angeles in the Southwest region of California down to South of Ensenada in Baja California, with the highest habitat suitability East of Tijuana, with values up to 0.84 (Figure 1C). Under an RCP 8.5 scenario (Figure 1C), an increase of probability of habitat suitability was observed between Tijuana and Ensenada, with values up to 0.99. Moreover, an additional hotspot emerged up North close to Fresno, California. 

### 3.3. Distribution Models for Potential Rodents’ Reservoirs

**Figure 2:**
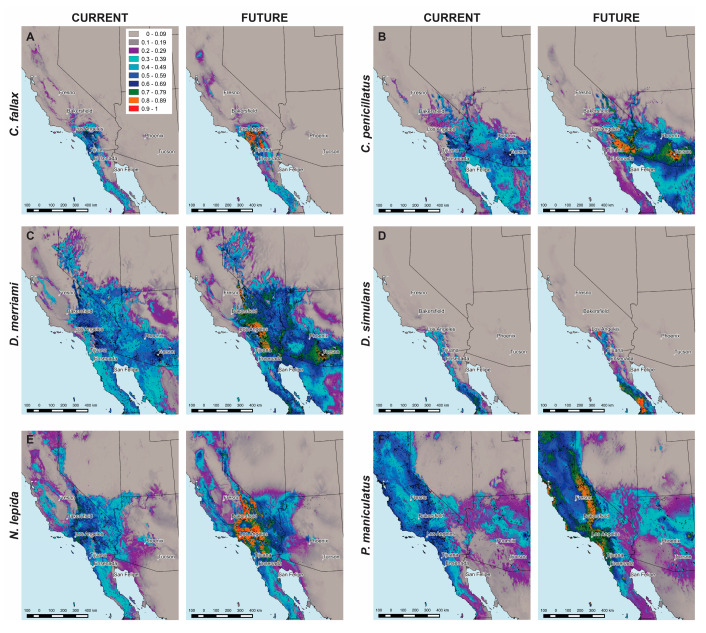
Distribution models for rodent species. Current environmental variables (first and third column) and those projected for 2070 based on a RCP 8.5 (second and fourth column) for (**A**) *C. fallax*, (**B**) *C. penicillatus*, (**C**) *D. merriami*, (**D**) *D. simulans*, (**E**) *N. lepida* and (**F**) *P. maniculatus*. Habitat suitability for each species is represented in 10 categories based on probability: from 0 to 0.09 (gray), from 0.1 to 0.19 (dark grey), from 0.2 to 0.29 (purple), from 0.3 to 0.39 (turquoise), from 0.4 to 0.49 (dark turquoise), from 0.5 to 0.59 (blue), from 0.6 to 0.69 (dark blue), from 0.7 to 0.79 (green), from 0.8 to 0.89 (orange) and from 0.9 to 1 (red). Black dots represent occurrence points of each rodent species downloaded from The Global Biodiversity Information Facility.

DM for the San Diego pocket mouse *C. fallax* showed a delimited range from Los Angeles in California to the South region of Baja California (Figure 2A). The highest values of habitat suitability were projected in Southern California and Northern Baja California (0.80). A similar pattern was observed under an RCP 8.5 scenario, although with a probability of habitat suitability close to 0.99. DM for the desert pocket mouse *C. penicillatus* projected habitat suitability in all three states with higher values of probability (0.77) in the southeastern region of California and Southern Arizona (Figure 2B). A similar pattern was observed under an RCP 8.5 scenario. However, higher values of habitat suitability (up to 0.97) were projected both in Arizona and California. DM for the Merriam’s kangaroo rat *D. merriami* (Figure 2C) projected broad habitat suitability in California, Arizona and Baja California, with values of 0.74. Under an RCP 8.5 scenario, a very similar pattern was observed, although with values of habitat suitability of 0.95. As for *C. fallax*, the DM for the Dulzura kangaroo rat *D. simulans* projected a more restricted suitable area in Southern California and in Baja California (Figure 2D), with greater suitability values in the South region of Baja California (values of 0.83). A similar pattern was observed under an RCP 8.5 scenario, although the probability of habitat suitability rose to 0.96 in Baja California and to 0.99 in California (East of Los Angeles). DM for the desert woodrat *N. lepida* projected suitable areas in all three states, with higher suitability in California with values of 0.87 (Figure 2E). Under an RCP 8.5 scenario, the probability of habitat suitability in California rose to 0.99. Finally, DM for the deer mouse *P. maniculatus* projected suitable areas in the three states with higher values (0.76) mostly in California (Figure 2F). Under an RCP 8.5 scenario, the probability of habitat suitability rose to 0.98.

### 3.4. Overlap of Environmentally Suitable Areas for All Species

**Figure 3:**
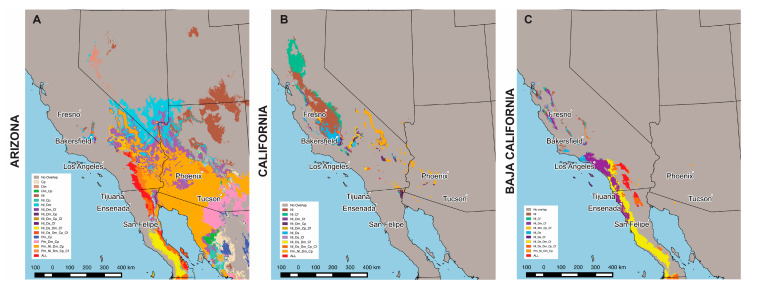
Overlaps of DMs for *Coccidioides* spp. and for six rodent species. Areas of distribution overlap between *Coccidioides spp*. DM, considering its statistically significant presence for Arizona (**A**), California (**B**), Baja California (**C**) and DM for each rodent species, considering their statistically significant presence for each state. In inset: Cp: *Chaetodipus penicillatus*, Dm: *Dipodomys merriami*, Nl: *Neotoma lepida*, Cf: *Chaetodipus fallax*, Ds: *Dipodomys simulans*, Pm: *Peromyscus maniculatus*.

In Arizona, the greatest overlap of environmentally suitable areas occurs for *Coccidioides* spp. and four rodent species, the two Heteromyidae species, *D. merriami* and *C. penicillatus*, and the two Cricetidae species, *P. maniculatus* and *N. lepida* (Figure 3A). Similarly, for Baja California, the greatest overlap occurs between *Coccidioides* spp. and four rodent species, in this case, *N. lepida* and three Heteromyidae species, *D. simulans, D. merriami* and *C. fallax* (Figure 3C). In addition, in Arizona and Baja California, the fungus DM coincided fully with the DM of the six rodent species only in delimited areas in south central California and North central Baja California. In contrast, in California, the largest area of overlap occurs between *Coccidioides* spp. and *N. lepida*, and between *Coccidioides* spp., *N. lepida* and *C. fallax* (Figure 3B).

### 3.5. DMs and CM Incidence in Arizona and California

Information regarding the incidence of CM reported in the counties of California and Arizona was superimposed to the DM maps for *Coccidioides* spp. (Supplementary Figure S3). For both Arizona and California, counties with reported greater incidence numbers overlap with DM regions of environmentally suitable habitat for *Coccidioides* using current variables and occurrence points (Supplementary Figure S3). In Arizona, Maricopa County, with the highest reported number of cases (87 cases per 100,000 population), overlaps with areas of high habitat suitability with values up to 0.78. For projected future scenarios, although considering current incidence reports, values of habitat suitability raised up to 0.96 in Maricopa and Pinal, both counties that have reported higher number of cases per 100,000 population in Arizona in the 2017–2018 period. In California, Kern County, with reports in 2016, the highest number of cases (251.7 cases per 100,000 population) overlaps with areas of high habitat suitability (with values up to 0.80). In projected future scenarios, although considering current incidence reports, values of habitat suitability raised up to 0.97 for Kern County.

## 4. Discussion

In this work, we have used Maxent and GIS to model current and future potential distribution of *Coccidioides* spp., the causal agent of Coccidioidomycosis or Valley Fever, in Baja California, Mexico, and in California and Arizona in the United States. More delimited distributions for the fungus than those reported previously with GARP were obtained [29]. This would reduce the areas for direct soil samplings and would facilitate the identification of potential hotspots.

Within the two genetically diverse species of *Coccidioides*, distinct populations have been identified: the San Joaquin Valley and San Diego/Mexico populations for *C. immitis*, and the Texas/South America, Mexico and Arizona populations for *C. posadasii* [46]. The limited gene flow detected among the populations suggests local adaptation [46], which is consistent with the geographically distinct populations previously reported [47]. Likewise, we show different geographical patterns identified by the SDMs. The DM for *Coccidioides* obtained using occurrence points for Arizona resembles the distribution of *C. posadasii* populations in Arizona and Mexico, whereas DM for *Coccidioides* obtained using occurrence points for California and Baja California resembles the distribution of *C. immitis* populations in San Joaquin valley and San Diego/Mexico. This suggests that *Coccidioides* spp. could have adapted to different local environments in the three states considered in this study.

For DM construction, based on Jackknife results and percentage of contribution of all environmental variables, sand percentage within the first 15 cm of soil was not important for any of the *Coccidioides* DMs. However, given the previous reported correlation between sandy alkaline soils and the fungus at a local scale [5,9], higher-resolution variables containing information on soil parameters could contribute to generate more accurate models, hence potentially providing us with finer scale variables to construct large-scale prediction maps to facilitate future samplings.

In soil heat physics, soil temperature is known to be dependent on various parameters, including meteorological conditions [48]. Furthermore, an increase in temperature affects negatively on soil moisture due to the increase of evaporation [49] and it has been reported that changes in air temperature of 1.6 °C and soil temperature (at 5 cm depth) of 0.5 °C can change fungal communities’ composition [50]. Soil temperature, as well as several factors including amount and timing of rainfall, available moisture, soil humidity and others, are considered to influence *Coccidioides* growth [9]. The use of global climate data to model the distribution of such a small fungal organism may cause some uncertainty dependent on geographical and study variables scales. However, diverse research groups are using these tools to model pathogens’ distribution such as dengue, malaria, Zika virus, leishmaniases vectors and *Batrachochitrium dendrobatidis*, a fungus that affects amphibians [31,32,51,52,53,54].

An increase in global temperature, atmospheric CO_2_, ozone, changes in humidity, rainfall and severe weather are projected for the coming decades by models of climate change, and climate fluctuation could lead to changes in fungal phenology, such as fruiting patterns [55,56]. The increase in habitat suitability under a future scenario was consistent for *Coccidioides* DMs of the three states. This would support the general trend of increased disease severity as well as changes in pathogen distribution that have been predicted for future conditions according to climate change models [57].

Each rodent species DM was evaluated based on its AUC values, considering the qualitative indices proposed previously [58]. *P. maniculatus* and *D. merriami* had the lowest AUC values. These lower model performance values are usually expected for species with broad distributions, which unlike species with narrow distributions often lack well-characterized niches, hence being in general harder to model [59].

In earlier studies, species of heteromyidae belonging to the *Chaetodipus* and *Dipodomys* genera (*C. penicillatus* and *D. merriami*) were reported in San Carlos, Arizona [25], and evidence of unidentified rodents was observed in Bakersfield, Wasco and Arvin in California, where *Coccidioides* spp. was detected from soil samples [60]. In the current study, the largest area of overlap in California occurs between *Coccidioides* spp. DM and that for *N. lepida*, the desert woodrat. Furthermore, this species DM overlapped with *Coccidioides* DM in the other two states. While there are no records of this rodent as a potential reservoir for *Coccidioides* in California or in Arizona, antibodies against *Coccidioides* were found in sera of *N. lepida* in specimens collected in Baja California [24]. This indicates that perhaps *N. lepida* could be a favorable reservoir in all states, while the other rodents contribute to the regional distribution of *Coccidioides* in each state. The study site Valle de las Palmas apparently presents unique components that sustain the presence of the six rodent species considered in this work and *Coccidioides* spp., making this a target site for further research on the interactions of the fungus and its potential hosts.

*Coccidioides* is able to persist for several years in soil, but it is also able to move and grow outside the endemic area, presumably driven by rodent or other animal populations’ movements [61]. Nevertheless, it is important to clarify that *Coccidioides* does not depend specifically on rodents for dispersion. Wind, water, other mammal hosts and anthropogenic activities can contribute to its transport, and fungal spores can be carried as well, along with dust, in bodies and digestive tracts of animals [9,62,63]. However, the endozoan hypothesis proposed by Taylor and Baker suggests that the fungus persists inside small mammals as spherules in granulomas, and when the host dies from either disseminated CM or other natural cause, hyphal growth begins. In that situation, given *Coccidioides* ability to digest animal protein, its growth is successfully sustained by the dead mammals’ carcasses, favoring the production of abundant arthroconidia [64]. Given the suggested importance of rodents for the fungus dissemination and our results, where CM incidence correlated with DMs, we propose that rodents’ distribution should be considered for future research. Sites with high probability of co-existence of the fungus and the rodents identified in the DMs should be sampled to reveal potential hotspots.

## 5. Conclusions

Our results indicated a spatial correlation (overlap) between sites with high values of habitat suitability in the DMs of the fungus and the incidence of CM. In all regions, there was a predominant overlap between the potential distribution of the fungus and areas where *N. lepida* or *C. fallax* were potentially distributed. The other rodent species are relevant to a local (state) scale. Finer ecological and molecular studies towards identifying the potential natural reservoirs or hosts for *Coccidioides* spp. are required to better characterize and understand its ecological niche, thus allowing to design directed samplings and to identify more positive sites. Altogether, our results suggest that both abiotic and biotic variables should be taken into account to implement surveillance programs to monitor the spread of the disease.

There are many concerns associated with global climate change. In this study, we focused on the potential distribution shifts of *Coccidioides* spp. DM projected for 2070 in Baja California, Mexico, and the South western United States, when a potential increase of 2–3 °C under high emission scenarios is projected [65]. The results indicate an increase of the probability of suitable habitat for *Coccidioides* spp. in certain areas, where it already resides, such as the Central Valley in California, and Maricopa in Arizona. This increase may lead to a rise in CM incidence in the population.

There is a strong need of improved sampling methods and finer scale environmental variables to obtain more accurate DMs. This may lead to identifying potential sites, where *Coccidioides* is more likely to be found in soil samples, which in turn would result in more occurrence data that could be included in robust and improved future DMs for *Coccidioides*.

## Supplementary Materials

The following are available online at https://www.mdpi.com/2309-608X/6/4/320/s1, Figure S1: Jackknife test carried out for all SDMs. Figure S2: Area under the receiver operating curve (AUC). Figure S3: DM for *Coccidioides* spp. and CM incidence in Arizona (**A**) and California (**B**), using current environmental variables (left), and those predicted for 2070 based on an RCP 8.5 scenario (right), Table S1: Environmental variables.
