# Supplementary material for: Of Mice and Fungi: Coccidioides spp. Distribution Models
Source: J Fungi (Basel). 2020 Nov 27;6(4):320. doi: 10.3390/jof6040320 (PMC7712536; doi:10.3390/jof6040320)
Supplement: Supplementary file 1 [file jof-06-00320-s001.zip › Supplementary Figures.docx]

**

**

Figure S1. Jackknife test carried out for all SDMs. Variables showing the highest gain when used in isolation (dark blue) are: (A) BIO10, BIO18 and BIO3 for *Coccidioides* in Arizona, California and Baja California, respectively, (B) BIO14 for *C. fallax*, (C) BIO1 for *D. merriami*, (D) BIO15 for *P. maniculatus,* (E) BIO18 for *N. lepida,* (F) BIO1 for *C. penicillatus* and (G) BIO4 for *D. simulans*. Variables whose gain decrease the most when they are omitted (light blue) are: (A) BIO15, BIO6 and BIO6 for *Coccidioides* in Arizona, California and Baja California, respectively, (B) BIO17 for *C. fallax*, (C) BIO19 for *D. merriami*, (D) BIO15 for *P. maniculatus,* (E) BIO15 for *N. lepida,* (F) BIO8 for *C. penicillatus* and (G) BIO5 for *D. simulans*.

**
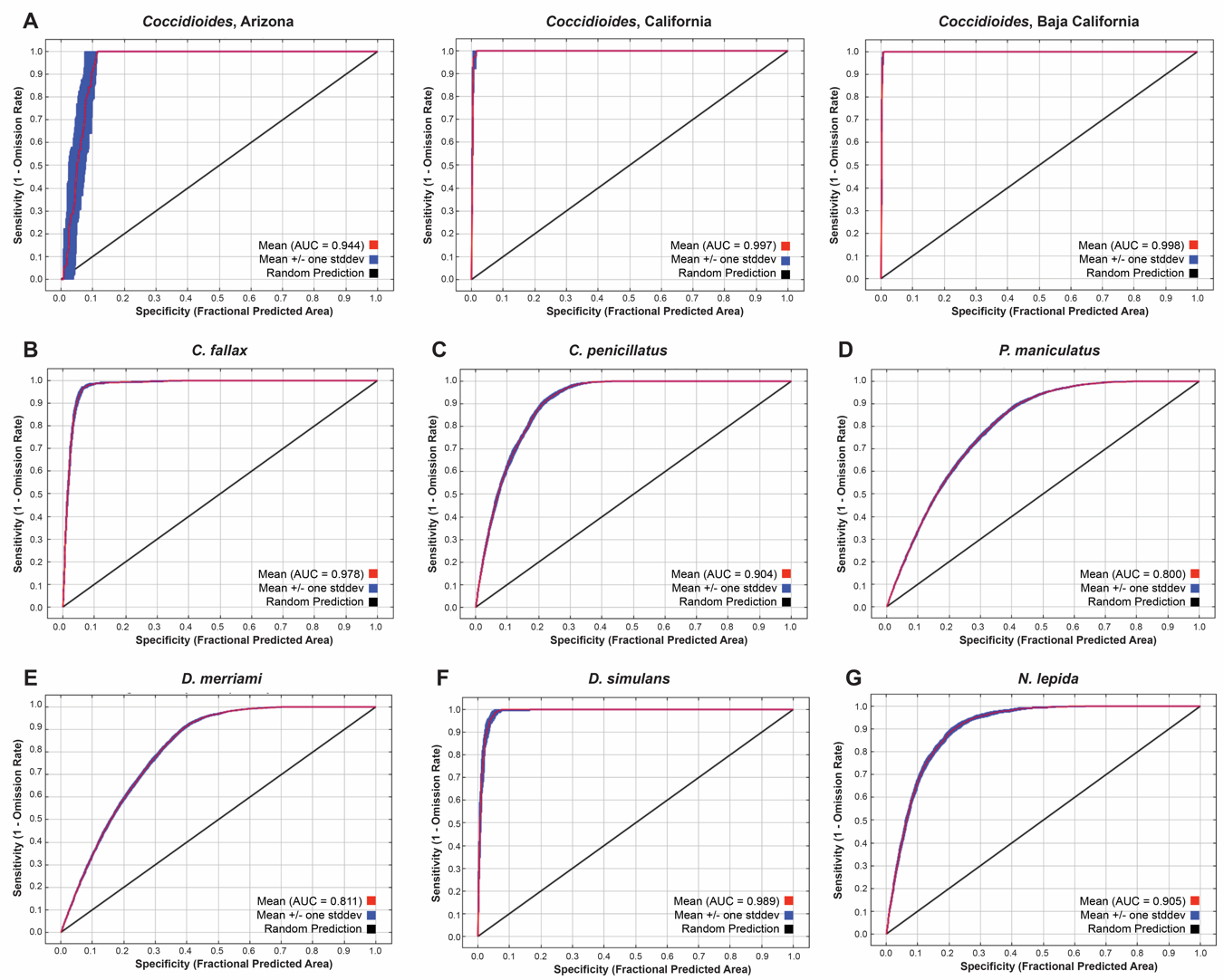
**

**Figure S2.** Area under the receiver operating curve (AUC). The red line represents the mean value for the 10 MaxEnt runs, ± standard deviation is represented by the blue area and AUC value for random prediction is represented by a black line. Mean AUC values ≥0.8 indicate good model predictions. AUC values for *Coccidioides* were 0.944 in Arizona (**A**), 0.997 in California (**B**), and 0.998 in Baja California (**C**). For rodents, AUC values were for *C. fallax* 0.978 (**D**), for *C. penicillatus* 0.904 (**E**), for *P. maniculatus* 0.800 (**F**), for *D. merriami* 0.811 (**G**), for *D. simulans* 0.989 (**H**), and for *N. lepida* 0.905 (**I**).


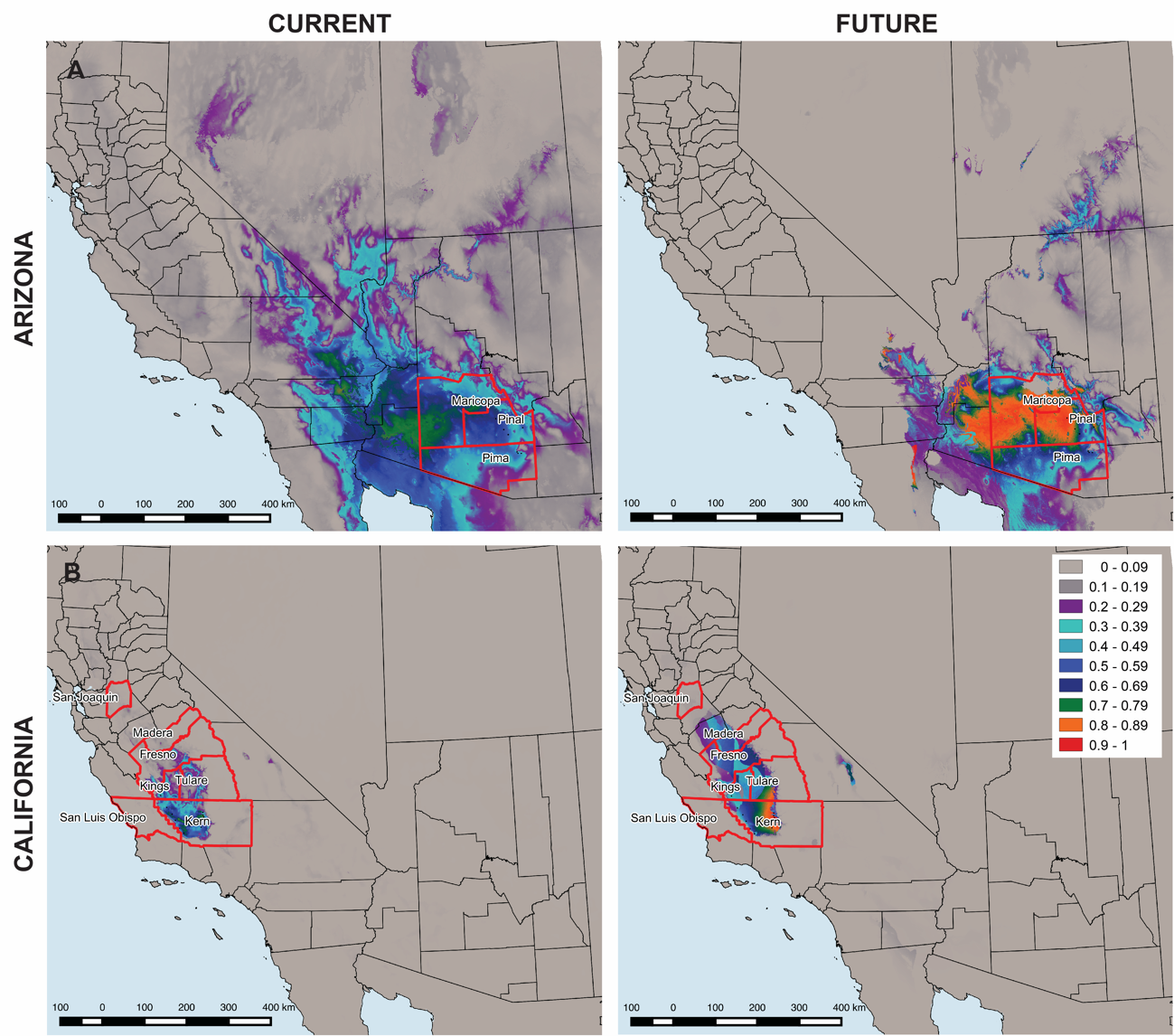


**Figure S3.** DM for *Coccidioides* spp. and CM incidence in Arizona (**A**) and California (**B**), using current environmental variables (left), and those predicted for 2070 based on an RCP 8.5 scenario (right). Counties delimited in red represent those with the highest number of cases per 100 000 population; California rates: 251.7 in Kern County and 54.5 in Fresno, Kings, Madera, San Joaquin, San Luis Obispo, and Tulare. Arizona rates: 87, 76.9 and 58.6 in Maricopa, Pinal and Pima County, respectively.
