# Supplementary material for: Of Mice and Fungi: Coccidioides spp. Distribution Models
Source: J Fungi (Basel). 2020 Nov 27;6(4):320. doi: 10.3390/jof6040320 (PMC7712536; doi:10.3390/jof6040320)
Supplement: Supplementary file 1 [file jof-06-00320-s001.zip › Table S1.docx]

Table S1. Environmental variables.

| Code | Environmental variable | Source |
| --- | --- | --- |
| **BIO1** | Annual Mean Temperature | Worldclim Version2 |
| **BIO2** | Mean Diurnal Range (Mean of monthly (max temp - min temp)) |  |
| **BIO3** | Isothermality (BIO2/BIO7) (*100) |  |
| **BIO4** | Temperature Seasonality (standard deviation *100) |  |
| **BIO5** | Max Temperature of Warmest Month |  |
| **BIO6** | Min Temperature of Coldest Month |  |
| **BIO7** | Temperature Annual Range (BIO5-BIO6) |  |
| **BIO8** | Mean Temperature of Wettest Quarter |  |
| **BIO9** | Mean Temperature of Driest Quarter |  |
| **BIO10** | Mean Temperature of Warmest Quarter |  |
| **BIO11** | Mean Temperature of Coldest Quarter |  |
| **BIO12** | Annual Precipitation |  |
| **BIO13** | Precipitation of Wettest Month |  |
| **BIO14** | Precipitation of Driest Month |  |
| **BIO15** | Precipitation Seasonality (Coefficient of Variation) |  |
| **BIO16** | Precipitation of Wettest Quarter |  |
| **BIO17** | Precipitation of Driest Quarter |  |
| **BIO18** | Precipitation of Warmest Quarter |  |
| **BIO19** | Precipitation of Coldest Quarter |  |
| **Soil3** | Sand content (50–2000 μm) mass fraction in% | SoilGrids v0.5.3 |

All rasters have a 30 arc-second resolution.
